# Canstatin represses glioma growth by inhibiting formation of VM-like structures

**DOI:** 10.1515/tnsci-2020-0176

**Published:** 2021-08-10

**Authors:** Yuqiang Ma, Tao Wu, Houjie Zhou, Guilu He, Yifei Li, Bocheng Wang, Qiang Guo, Baodong Chen, Weiping Li

**Affiliations:** Neurosurgery Department, Peking University Shenzhen Hospital, 1120 Lianhua Road, Futian District, Shenzhen 518035, People’s Republic of China; GuangDong 999 Brain Hospital, Guangzhou 510510, People’s Republic of China; Neurosurgery Department, Shenzhen Second People’s Hospital, Sungang Road, Futian District, Guangzhou 510510, People’s Republic of China

**Keywords:** glioma, canstatin, vasculogenic mimicry, U87, VEGF, HIF-1α

## Abstract

Vasculogenic mimicry (VM) is different from classical tumor angiogenesis and does not depend on endothelial cells. VM is closely related to the prognosis of various cancers. Canstatin was first identified as an endogenous angiogenesis inhibitor. In the present study, the inhibitory effect of canstatin on VM formation was evaluated. Human glioblastoma cell lines U87 and U251 were letivirally transduced to overexpress canstatin gene or GFP as control. *In vitro* assays showed that canstatin overexpression reduced the tube formation of U87 and U251 cells in Matrigel. A xenograft glioma model was created by subcutaneous injection of lentivirally modified U87 cells into nude mice. The results of *in vivo* experiments showed that canstatin gene introduction inhibited the growth of glioma xenografts. In tumor xenografts overexpressing canstatin, U87-mediated formation of VM-like structures and VM-related VEGF (vascular endothelial growth factor) expression were remarkably reduced. Canstatin overexpression also decreased the phosphorylation of Akt and reduced the expression of Survivin *in vitro.* In addition, HIF-1α production and MMP-2 secretion were decreased by canstatin overexpression. Therefore, these results suggested a protective role of canstatin during VM-like structure formation of glioma probably via inhibiting signaling pathways inducing vasculogenic mimicry.

## Abbreviations

VM: vasculogenic mimicry
PAS: periodic acid–Shiff
VEGF: vascular endothelial growth factor
AKT: protein kinase B
HIF-1α: hypoxia-inducible factor-1α

## 1 Introduction

Glioma is a primary tumor of the nervous system. The average survival time of patients with anaplastic glioblastoma is only 12–15 months [1]. Studies have shown that high-grade gliomas can obtain rapid blood supply even if they do not have vascular endothelial formation [2]. Vasculogenic mimicry (VM) is a neovascularization phenomenon that was first reported in melanoma models [3]. It has been established that VM is independent of endothelial blood vessels and formed by deregulated tumor cells. These tumor cell-lined vessels are positive for periodic acid–Shiff (PAS) staining [3]. Current studies have shown that VM exists in various malignant tumors such as lung cancer [4], hepatocellular carcinoma [5], and malignant glioma [6]. Most studies suggest that VM may promote a poor prognosis in cancer patients.

Canstatin is a fragment of the noncollagenous NC1 domain of type IV collagen alpha2 chain [7]. It exists at a higher abundance than endostatin, a 20 kDa C-terminal fragment of collagen XVIII and the anti-angiogenic protein in the isolated basement membrane [7]. Studies have confirmed that canstatin inhibits the migration and proliferation of vascular endothelial cells *in vitro* and can also induce apoptosis in endothelial cells and inhibit tumor growth *in vivo* [7,8]. However, the effect of canstatin on tumor cell-mediated VM formation has not been clarified.

Vascular endothelial growth factor (VEGF) is involved in neovascularization and VM formation during tumorigenesis [9,10]. The co-expression of VEGF and survivin, an apoptosis inhibitor, has been reported in various types of cancers [11,12]. It was shown that survivin expression is upregulated by VEGF-induced Akt activation [13]. A previous study demonstrated that canstatin can inhibit Akt activation and induce apoptosis in endothelial cells [14]. Another primary signaling molecule inducing VM is hypoxia-inducible factor-1α (HIF-1α). Overexpression of HIF-1α protein has been detected in malignant tumors and precancerous lesions, but not in normal tissues and benign lesions [15]. The previous study reported that HIF-1α, as a transcription factor, can activate genes such as MMP-2, driving vasculogenic mimicry [16]. It remains to be determined whether canstatin participates in VM formation by regulating VEGF/Akt/survivin or HIF-1α/MMP-2 pathway.

Therefore, in the present study, a preclinical model was established by implantation of nude mice with U87 glioma cells that were manipulated by lentivirus-mediated canstatin overexpression. VM-associated signaling pathway and mediators, including VEGF, Akt, survivin, and HIF-1α, were examined to investigate the underlined mechanism.

## 2 Material and methods

### 2.1 Animal models

BALB/c nude mice (aged 5 weeks, 20–25 g, male) were purchased from Slac Laboratory Animal Corporation (Shanghai, China). Mice were kept under pathogen-free conditions at room temperature (21–25°C) in 12 h–12 h light–dark cycle.

**Ethical approval:** This research adhered to the Principles of Laboratory Animal Care (NIH publication No 85–23, revised 1985). All experimental protocols described in this study were approved by Animal Care and Use Committee of Peking University Shenzhen Hospital (approval No. 2019-003).

### 2.2 Reagents and antibodies

The primary antibodies were purchased from Proteintech Group Inc. (IL, USA), including CD31 rabbit polyclonal antibody (#11265-1-AP), VEGF rabbit polyclonal antibody (#19003-1-AP), Akt rabbit polyclonal antibody (#10176-2-AP), Akt-phospho-S473 mouse monoclonal antibody (#66444-1-Ig), HIF-1α rabbit polyclonal antibody (#20960-1-AP), and GAPDH mouse monoclonal antibody (#60004-1-Ig). The primary antibody against canstatin (#DF3550, rabbit polyclonal antibody) was obtained from Affinity Biosciences Inc. (OH, USA). The primary antibody against survivin rabbit polyclonal antibody (#GB11177) was purchased from Servicebio Inc. (Wuhan, China). The HRP-linked goat anti-mouse IgG secondary antibody (#SA00001-1) was purchased from Proteintech Group Inc. (IL, USA). The HRP goat anti-rabbit IgG secondary antibody (#7074) was purchased from Cell Signaling Technology Co. (Danvers, MA, USA). The primers used in this study were synthesized by Takara Biotechnology Co., Ltd (Dalian, China).

### 2.3 Cell lines and cell cultures

HEK293 cells were obtained from CCTCC (China Center for Type Culture Collection, Wuhan, China). Human glioma cell lines U87 (No. 3111C0001CCC000208) and U251 (No. 3111C0001CCC000058) were obtained from the Institute of Biochemistry and Cell Biology (Shanghai Institutes for Biological Sciences, Chinese Academy of Sciences, Shanghai, China). Cells were cultured in Dulbecco’s modified Eagle’s medium (DMEM; Life Technologies, MD, USA) and supplemented with 10% fetal bovine serum (FBS; Life Technologies), 100 units per milliliter of penicillin (Life Technologies), and 100 µg/mL of streptomycin (Life Technologies) at 37°C in a humidified 95% air and 5% CO_2_ atmosphere.

### 2.4 Lentiviral vector production and titration

Briefly, 6 × 10^6^ HEK293 cells were seeded in 10 cm^2^ plates. After 24 h, cells were transfected at 60–80% confluence using Lipofectamine™ 2000 (Life Technologies) and mixture of plasmids, typically pCDH-EGFP or pCDH-Canstatin-EGFP, Delta-8.9 lentiviral envelope plasmid (BioVector NTCC Inc., Beijing, China), and pLP-VSVG lentiviral packaging plasmid (Invitrogen; 15:12:8). The medium was changed after 24 h. Culture medium was then collected 48, 72, and 96 h after transfection. After collection, the lentivirus-containing medium was passed through 0.45 μm filters and stored at −80°C.

The functional titer was determined by the flow cytometric analysis for EGFP expression following infection of HEK293 cells. Briefly, 6 × 10^5^ HEK293 cells were infected with lentiviruses plus 8 μg/mL polybrene (Sigma-Aldrich). After 48 h, EGFP expression in the infected cells was determined by using the FACSCalibur platform (BD Biosciences, CA, USA), and data were analyzed by Flowjo software. Titers were calculated from virus dilutions, where 10–20% of the cell population was EGFP positive.

### 2.5 Glioma cell lentiviral transduction

Concentrated lentivirus was obtained by centrifugation at 1,200 *g* for 40 min at 32°C. U87 or U251 cells were loaded onto six-well plate (1.5 × 10^5^ per well) and incubated overnight. Cells were transduced with lentivirus supernatant overnight at MOI 0.5 or 5 (based on HEK293 transduction units). The lentiviral transduction was repeated 8 h later. After 96 h, transduced cells were analyzed for EGFP expression by flow cytometry (FACSCalibur, BD Biosciences). Images were taken by using the IX71 fluorescence microscope (Olympus Co., Otsu, Japan). If not otherwise specified, the assays for U87 or U251 cells in this study were performed after 72–96 h incubation after lentiviral transduction when the cell confluency was about 85%.

### 2.6 Xenograft glioma model establishment and tumor volume measurement

Nude mice were randomly assigned to either the control or the canstatin group for the respective treatment regimen. Each group at each time point had eight mice. Xenograft glioma model was created by subcutaneously injecting 5 × 10^6^ human U87 glioma cells, which were lentivirally transduced to overexpress lentivirus vector (Vector group) or canstatin (canstatin group). Cells were suspended in 50 µL of saline at 3 mm to the right back of the mice. Tumor volume was measured every 7 days after implantation, and at least three tumor sizes were recorded. Each group of nude mice was put together and photographed with a ruler. Two weeks after tumor implantation, animals underwent analysis followed by euthanasia by slow (20–30% per minute) displacement of chamber air with compressed CO_2_. Heartbeat and respiration were checked to verify death. The samples were then collected for further analysis.

### 2.7 Immunohistochemical staining

CD31 endothelial marker and PAS dual staining (CD31-PAS) were examined for the existence of VM. Mice glioma tissue samples were fixed with 4% formaldehyde, embedded in paraffin, and sectioned into serial section (4 μm thick per nude mice). The sections were deparaffinized in xylene, hydrated, and boiled in EDTA antigen-unmasking solution. When cooled to room temperature, slides were incubated in peroxide at room temperature for endogenous peroxidase ablation. After being blocked with goat serum, slides were stained with a CD31 primary antibody overnight at 4°C. The slides were then washed with PBS and incubated with a secondary antibody at room temperature for 10 min. For visualization, a DAB horseradish peroxidase color development kit (Beyotime Inc., China) was used according to the manufacturer’s instructions. Then, the slides were exposed to the periodic acid solution for 10 min, incubated with Schiff solution for 10 min in the dark, and counterstained with Mayer’s hematoxylin (Solarbio Life Sciences Inc., China). Immunohistochemical staining with VEGF antibody was performed with the immunohistochemistry (IHC) assay kit (Thermo, USA). Pannoramic scanner (3DHISTECH Ltd., Budapest, Hungary) was used to scan the whole tissue images of serial sections, reducing the chance of biasness by taking pictures of specific areas of interest. Quantification of the images was done by HistoQuant™ software (3DHISTECH Ltd.).

### 2.8 Tube formation assay

A 96-well plate was precoated with 50 μL of Matrigel (Corning, Cat #354230) in each well and preincubated at 37°C for 1 h. U87 and U251 cells (2 × 10^4^/well) were resuspended with serum-free medium and loaded on the top of Matrigel. Following incubation at 37°C for 5 h, each well was analyzed directly under a microscope (IX71 Olympus Inverted Fluorescence Phase Contrast Microscope) with 10× phase contrast. Tubules in each field were imaged, and an average of tubules from three to five random fields in each well was quantified by the Angiogenesis Analyzer plug-in of Image J software version 1.51k. Three replicates were used in the tube formation assay.

### 2.9 CCK8 assay

U87 and U251 cells were prepared as described earlier and seeded in a 96-well plate (2,000 cells per well). After incubation at 37℃ for 2, 5, 12, and 24 h, the culture supernatant was removed. Cell viability was detected by adding 100 μL fresh DMEM and 10 μL CCK-8 (Cell Counting Kit-8) solution into each well followed by incubation of 3 h. The absorbance was tested at 450 nm using the plate reader (Tecan Group Ltd., Switzerland).

### 2.10 Apoptosis analysis

The transduced U87 and U251 cells overexpressing control vector or canstatin were trypsinized with 0.25% trypsin-EDTA (Life Technologies) and washed once in PBS with 2% FBS. Cells were then collected and stained with 10 μL Annexin V-APC (Life Technologies). After incubation in the dark for 10 min at room temperature, stained cells were analyzed on a flow cytometer (Beckman Coulter, Inc., Fullerton, CA, USA). ModFit program (Verity Software House Inc., USA) was used to analyze the proportion of apoptotic cells.

### 2.11 RNA isolation, reverse transcription, and quantitative PCR (qRT-PCR)

qRT-PCR analysis was performed as described previously [17]. Briefly, cells were seeded in a six-well plate and incubated overnight. After transduced as described earlier, U87 and U251 cells, respectively, overexpressing vector (control group) and canstatin (Canstatin group) were washed by PBS once after 72–96 h incubation. Cells were then collected by adding TRIzol Reagent (Invitrogen, Carlsbad, CA, USA) with 0.5 mL per well. Total RNA was extracted according to the manufacturer’s instructions. cDNA was prepared by using the ReverTra Ace qPCR-RT Kit (Toyobo, Osaka, Japan). PCR amplification was performed on StepOne Plus (Thermo Fisher Scientific, Waltham, MA, USA) as follows: 95°C for 10 min, and 40 cycles of 95°C for 10 s, 60°C for 15 s, followed by 72 for 20 s. The primers were designed as follows: canstatin (forward, 5′-TAAAGAGGAGCGCGACAGAT-3′; reverse, 5′-CTGTTGCCTTGCTGTCCTTT-3′), GAPDH (forward, 5′-TCAAGAAGGTGGTGAAGCAGG-3′; reverse, 5′-TCAAAGGTGGAGGAGTGGGT-3′). Melting curve analysis was used to assess the quality of the primers (Figure S1). Comparative CT (ΔΔCT) was used as the quantitation method for the qRT-PCR data analysis. Relative expression of Canstatin in control and Canstatin group were compared when data were normalized to GAPDH. Triplicate experiments and three independent qRT-PCR measurements were performed to obtain each sample ± standard deviation (SD).

### 2.12 Western blot

U87 cells were seeded in the six-well plates and incubated overnight. After transduced as described previously, U87 cells overexpressing vector (control group) and canstatin (canstatin group) were collected after 72–96 h incubation following lentiviral transduction when the cell confluency was about 85%. Cells were lysed with 250 µL RIPA buffer (WEIAO BioTech Co. Ltd, Shanghai, China) with 1 mmol/L PMSF (phenylmethylsulfonyl fluoride), a widely used protease inhibitor, on ice for 10–20 min. The supernatant extracts were quantified by using the BCA protein assay kit (Beyotime Inc.). The same amount (30 µg) of proteins were loaded on 10% SDS/PAGE gels and transferred to a piece of the PVDF membrane (0.45 µM). After blocking with 5% skim milk in TBST for 60 min, the membrane was incubated with the primary antibodies overnight at 4℃. Antibodies were diluted as suggested by the manufacturer’s instructions. Specifically, the primary antibody against canstatin was diluted at 1:500. Akt rabbit polyclonal antibody and Akt-phospho-S473 mouse monoclonal antibody were diluted at 1:1,000. HIF-1α rabbit polyclonal antibody was diluted at 1:200. GAPDH mouse monoclonal antibody was diluted at 1:20,000 and used as the loading control. Membranes were washed with the TBST buffer and incubated with horseradish peroxidase (HRP)-conjugated secondary antibodies for 1 h at room temperature. The chemiluminescence detection kit (Thermo Fisher Scientific, Waltham, MA, USA) was used to detect the protein signals with an X-ray film (Fuji Film, Tokyo, Japan). Quantification of western blots was analyzed with Image J software. The expression levels of each protein were normalized by that of the corresponding GAPDH. Three independent western blot experiments were performed to obtain each sample ± standard deviation (SD).

### 2.13 Enzyme-linked immunosorbent assay (ELISA)

Lentivirally transduced U87 cells were prepared as described earlier, and cell supernatants were collected at 4°C. The secretion of MMP-2 and MMP-9 was measured by using the MMP-2 ELISA kit (ml058669-2. MLBio, Shanghai, China) and the MMP-9 ELISA kit (ml058617-2, MLBio), respectively, according to the manufacturer’s instructions.

### 2.14 Statistical analysis

Data were expressed in mean ± standard deviation (SD) unless otherwise stated. Statistical analyses were performed using Student’s *t* test to analyze the difference between the means of two groups. Any *p* value less than 0.05 was considered significant.

## 3 Results

### 3.1 Canstatin overexpression reduced tube formation *in vitro*

**Figure 1:**
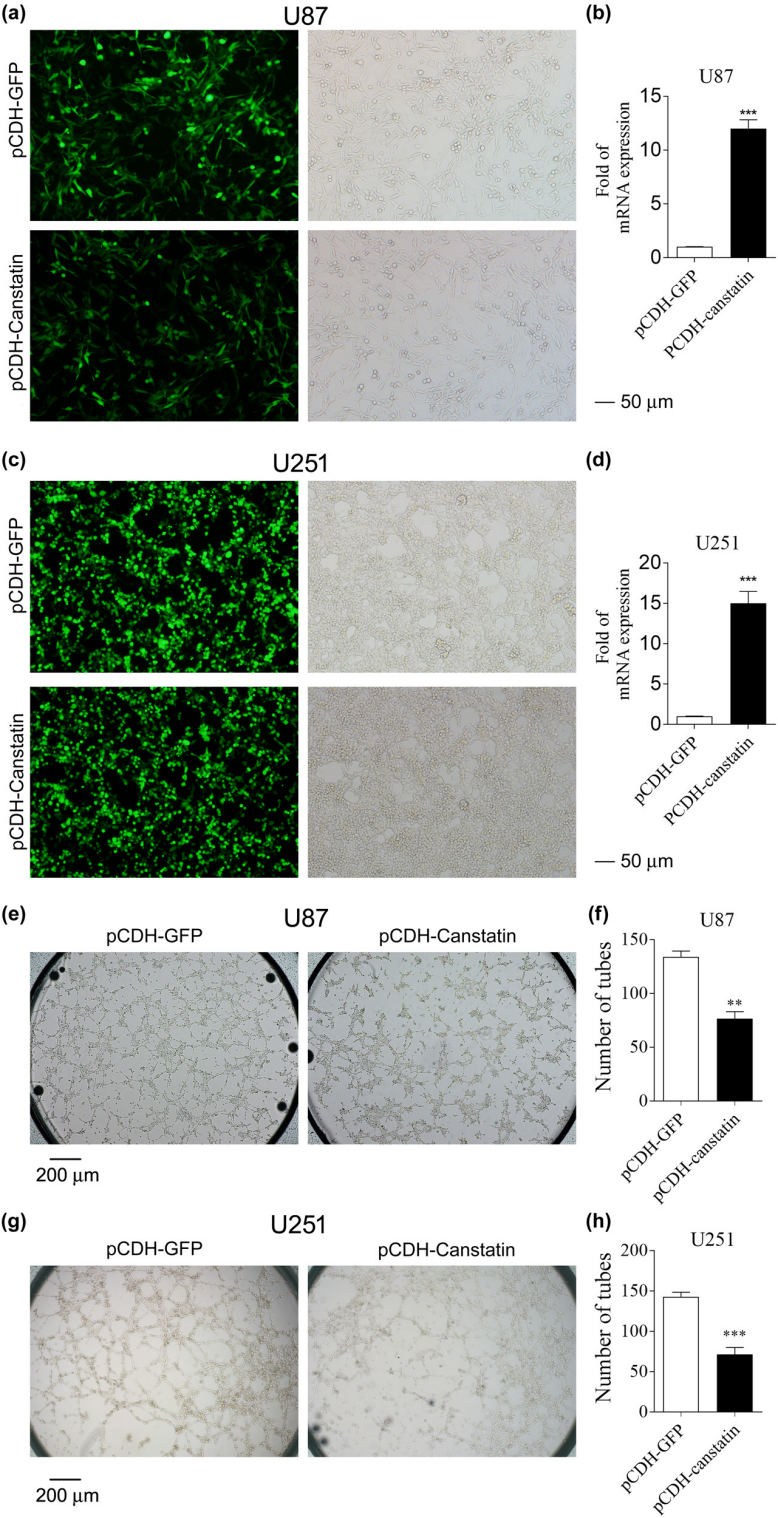
Vascular tube formation *in vitro.* Fluorescent images of U87 (a) and U251 (c) cells transferred with pCDH-EFGP and pCDH-Canstatin-EGFP. Scar bars, 50 μm. Quantification of mRNA expression levels of canstatin in U87 (b) and U215 (d) cells. Tube formation was measured with U87 (e) and U251 (f) cells expressing lentiviral pCDH vector or pCDH-canstatin. Tubules were imaged using phase contrast. A representative of three to five fields is shown. Scale bars, 20 μm. Quantification of tubules from three to five random fields in each well planted with the same amounts of U87 (g) and U251 (h) cells. Values are represented as means ± SD, *n* = 4 each group, *p < 0.05, **p < 0.01, and ***p < 0.005.

An *in vitro* tube formation assay was performed to investigate the effect of canstatin on the remodeling of the vasculogenic activity of glioblastoma cells. Canstatin-overexpressing U87 and U251 cells were obtained by using the lentiviral vectors system (Figure 1a–d). The same amounts of U87 or U251 cells were seeded on Matrigel. As expected, glioblastoma U87 and U251 cells could form vascular tubules with capillary-like structures (Figure 1e and f). Compared to the U87 cells transduced with lentivirus-based vector only, U87 cells overexpressing Canstatin formed significantly less-robust vascular tubules and cord-like structures (*p* < 0.05; Figure 1e and g). Similar results were observed when U251 cells were used (Figure 1f and h), indicating an inhibitory effect of canstatin in vascular tubules formation of glioblastoma. The CCK8 assay showed that canstatin overexpression significantly inhibited cell proliferation of U87 and U251 (Figure S2a and b). Apoptosis analysis suggested that overexpression of canstatin induced a higher proportion of apoptotic U87 and U251 cells (Figure S2c–f, *p* < 0.05). These results suggested that Canstatin might inhibit glioma tube formation by enhancing apoptosis.

### 3.2 Decreased size of tumors in response to canstatin gene introduction

**Figure 2:**
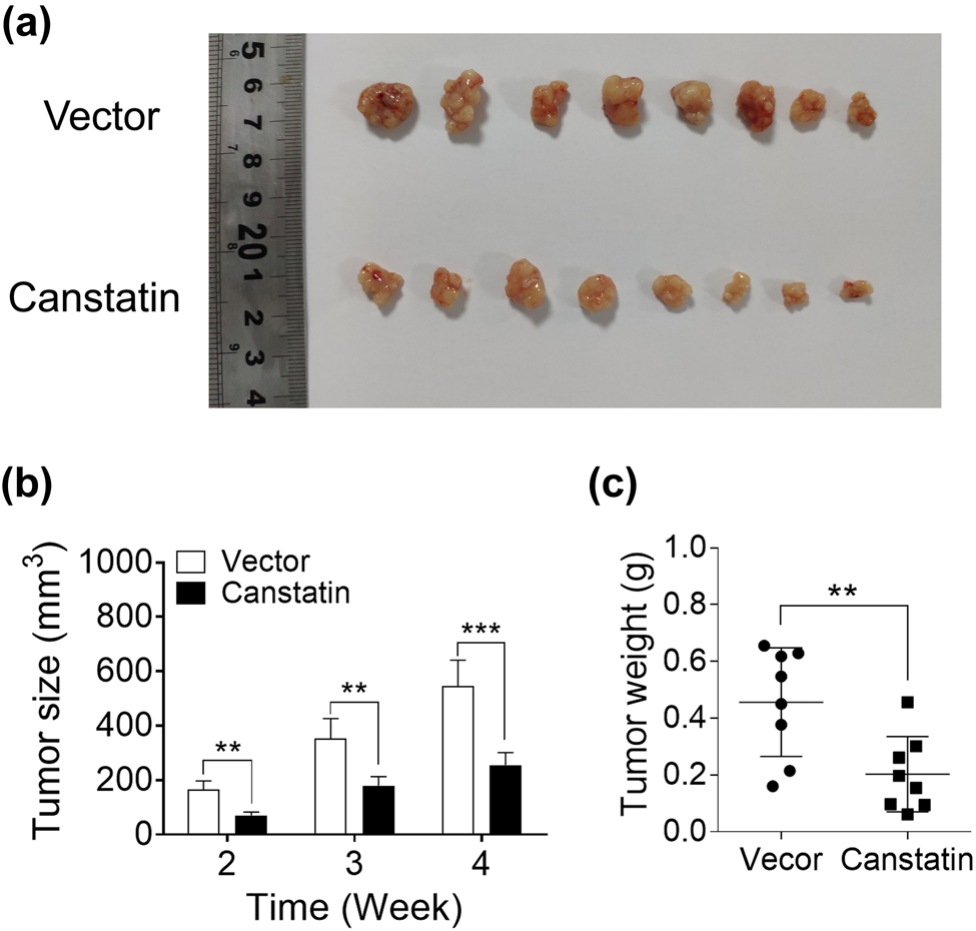
Canstatin overexpression inhibits xenograft tumor growth in nude mice. (a) Canstatin gene overexpression results in the suppression of tumor growth. Tumor size was calculated as (length × width^2^)/2 weekly for 4 weeks. (b) After 4 weeks of treatment, all mice were sacrificed, and the tumors were photographed. (c) The weights of tumors were measured. Values are represented as means ± SD, *n* = 8 each group, *p < 0.05, **p < 0.01, and ***p < 0.005.

Established xenograft tumor models in nude mice were used to test the effects of canstatin gene introduction to inhibit tumor growth. The nude mice were transplanted with U87 tumor cells expressing either lentivirus-based vector (vehicle group) or lentiviral vector-containing canstatin cDNA (canstatin group). All of the eight mice receiving control U87 cells developed tumors within 3 weeks (Figure 2a). In contrast, tumorigenesis was significantly inhibited in mice bearing canstatin-expressing U87 cells. These tumors were ∼50% smaller than those observed in the vehicle group (Figure 2a and b). Four weeks after transplantation, the final volumes of tumor xenografts in mice were 545.05 ± 52.34 mm^3^ (vector group) and 255.67 ± 37.35 mm^3^ (canstatin group; Figure 2b, *n* = 8 per group, *p* < 0.005). The tumor weights were 0.46 ± 0.23 g (vector group) and 0.20 ± 0.16 g (canstatin group; Figure 2c and d, *n* = 8 per group, *p* < 0.01). The maximum tumor diameters were 11.7 (vector group) and 11.0 mm (canstatin group). The maximum percentages of tumor weight per total body weight were 4.85% (vector group) and 3.51% (canstatin group). All mice were euthanized at the end of week 4. During 4 weeks of treatment, mice appeared healthy with no signs of wasting, and none of the mice died.

### 3.3 Attenuated VM formation following treatment with canstatin overexpression

**Figure 3:**
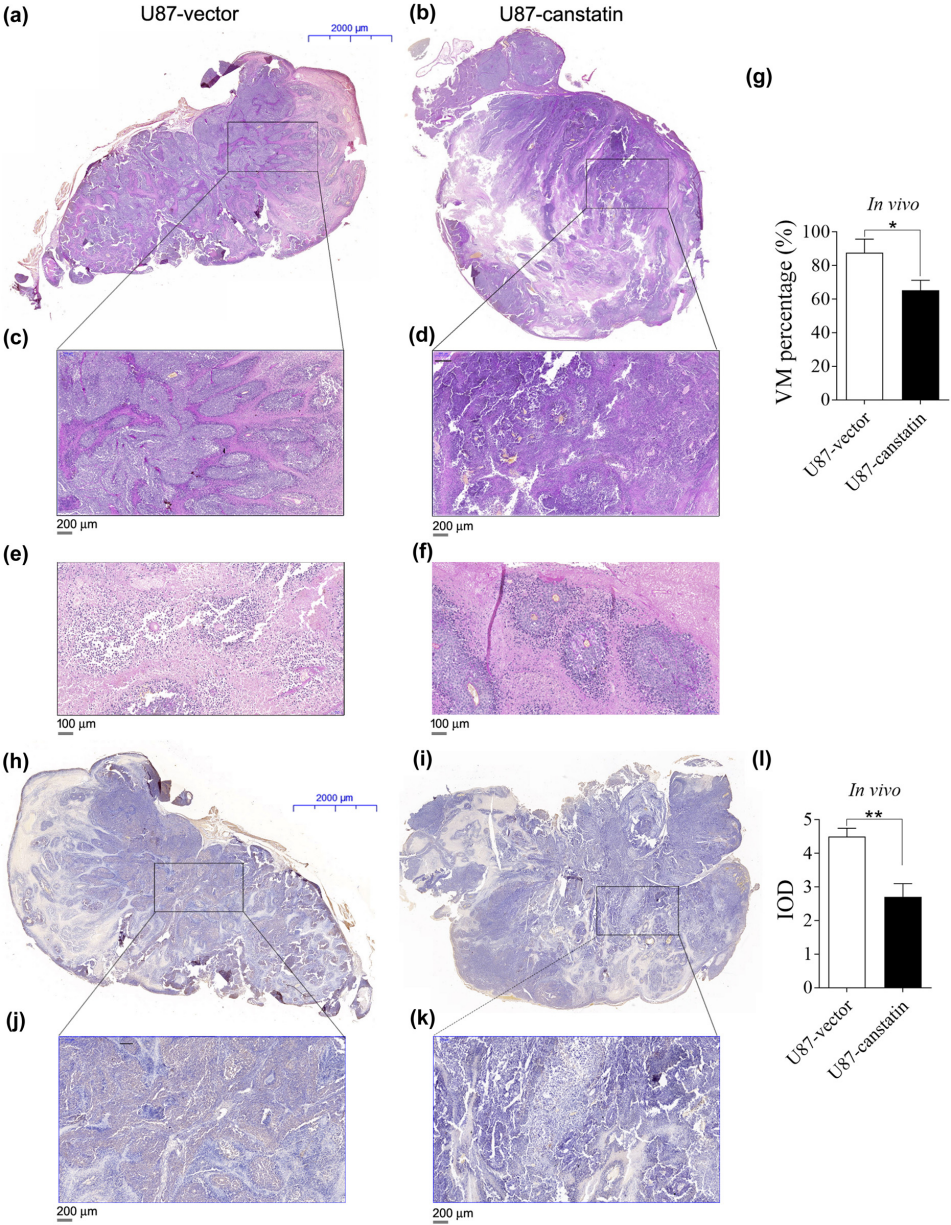
Immunohistochemistry of VM and VEGF in U87 xenografts. Panoramic views of the tumor xenografts expressing lentiviral pCDH vector (a) or pCDH-Canstatin (b). Tumor specimens were subjected to staining of both PAS and CD31. Scale bars, 2,000 μm. (c and d) Enlarged views of the central area of the xenografts shown in (a and b). Scale bars, 200 μm. Representative VM (arrows, CD31^−^/PAS^+^) in U87 xenografts expressing lentiviral pCDH vector (e) or pCDH-canstatin (f). Scale bars, 100 μm. (g) Quantification of VM ratios in the tumor xenografts. (h and i) Panoramic views of VEGF expressions in U87 xenografts. Scale bars, 2,000 μm. (j and k) Enlarged views of part of the xenografts that are shown in panels. (h and i) Scale bars, 200 μm. (l) The IOD of VEGF was significantly higher in the canstatin group than in the negative control. Values are represented as means ± SD, *n* = 4 each group, *p < 0.05, **p < 0.01, and ***p < 0.005.

The tumor xenografts (*n* = 4 per group) were collected and prepared as paraffin-embedded specimens. Serial sections (4-μm thick per nude mouse) were double stained by PAS and CD31 antibodies to detect the presence of VM (Figure 3a and b). PAS-positive and CD31-negative channels were deemed as VM (Figure 3c–f). A panoramic view of the tumor xenografts showed that the decrease in tumor size was consistent with a decrease in the counts of VM (Figure 3a–f). Among 16 serial sections, the VM ratio was counted by the percentage of VM vessels/total vessels per section. VM-positive rates were 87.6 ± 16.3% in control xenografts and 65.2 ± 12.1% in canstatin overexpression xenografts (*p* < 0.05; Figure 3g).

VM formation is related to the expression of vascular endothelial growth factor (VEGF). VEGF protein expression was also evaluated in additional serial sections by immunohistochemistry assay. Results showed a remarkably higher expression of VEGF at the peripheral part compared to that of the central area of xenografts (Figure 3h–k). The integrated optical density (IOD) of VEGF was significantly lower in the canstatin group than in the negative control group (Figure 3l, *p* < 0.001). These *in vivo* data suggest that the decreased tumor size correlates with the incidence of vasculogenic mimicry.

### 3.4 Canstatin overexpression decreased the phosphorylation of Akt and expression of survivin *in vitro*

**Figure 4:**
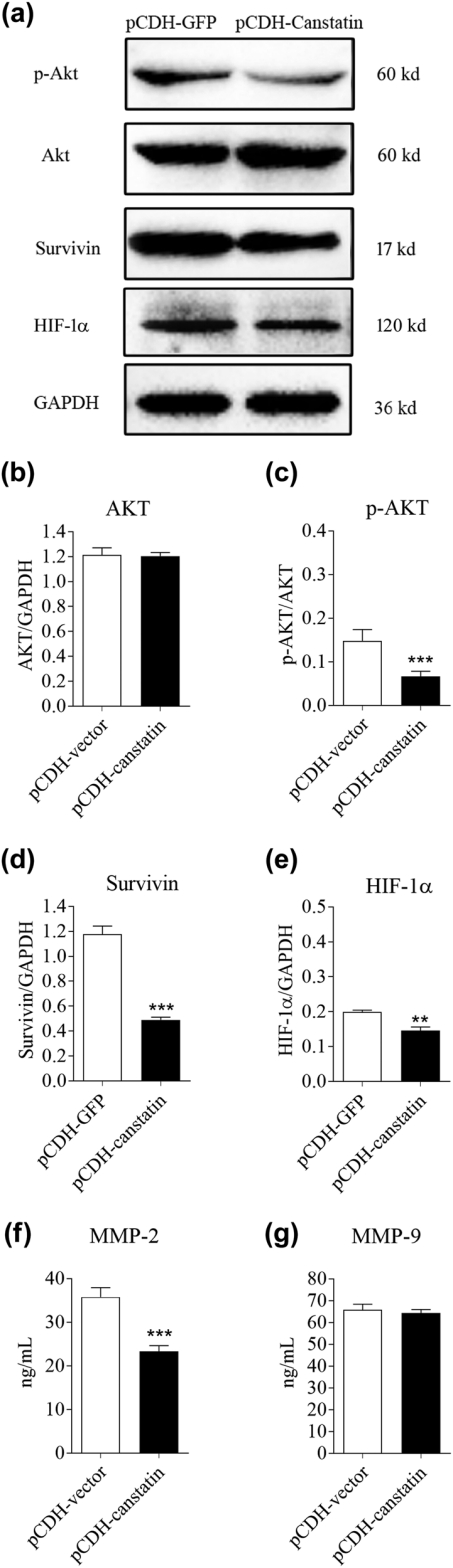
Canstatin overexpression suppressed the activation of Akt and decreased the expression of survivin *in vitro.* (a) Western blotting analysis of total Akt (Akt), phosphorylated Akt (p-Akt), survivin, and HIF-1α protein expression in U87 cells transduced with either lentiviral pCDH vector or pCDH-Canstatin. GAPDH was used as a loading control. (b–e) Relative quantification of Akt, p-Akt, survivin, and HIF-1α protein expression was determined. (f and g) ELISA analysis of MMP-2 and MMP-9 secretion in control and U87 cells overexpressing canstatin. Values are represented as means ± SD, *n* = 3 each group, *p < 0.05, **p < 0.01, and ***p < 0.005.

VEGF and its downstream signaling molecules, such as Akt, have been implicated in VM [9,10]. Our *in vivo* results showed that canstatin overexpression reduced the expression of VEGF in xenograft tumors (Figure 2h–l). To further investigate the mechanisms involved in this process, we analyzed the expression and activation of Akt by western blot analysis. Results showed that the expression of total Akt was not affected (Figure 4a and b), while the phosphorylation of Akt was significantly reduced by canstatin transfection into U87 cells (*p* < 0.005; Figure 4a and c). Survivin is overexpressed in an Akt-dependent manner in various human cancers [18]. Western blot analysis also revealed that canstatin overexpression remarkably decreased survivin expression in U87 cells (*p* < 0.005; Figure 4d). Therefore, canstatin may inhibit glioma-induced VM formation through suppressing the VEGF/Akt/surviving pathway.

### 3.5 Canstatin overexpression inhibited HIF-1α/MMP-2 VM pathway

In addition, we analyzed the expression of HIF-1α, one of the primary mediators of vasculogenic mimicry. Western blot results showed that upregulation of canstatin by lentiviral-mediated transfection increased HIF-1α protein levels in both U87 cells (Figure 4e, *p* < 0.005) and xenograft tumors (Figure S3). These data indicated that canstatin might play a role in repressing VM formation by reducing VM-associated signal transducer and transcriptional factor. The secretion of pro vasculogenic mimicry protein MMP-2 was also tested. The result showed that overexpression of canstatin increased MMP-2 secretion but not MMP-9 in U87 cells (Figure 4f and g).

## 4 Discussion

Canstatin has been identified as an endogenous antitumor molecule and is capable of inhibiting angiogenesis [7]. Angiogenesis is an important process in tumor invasion and metastasis, and it is dependent on the proliferation and capillary formation of vascular endothelial cells [19]. It has been established that canstatin treatment, such as recombinant canstatin application, can effectively inhibit the tumor growth mainly through its effective antiangiogenic effects [20]. However, whether canstatin regulates tumor VM has not been clarified.

In the present study, we first demonstrated that the glioma VM number could be decreased by canstatin gene overexpression *in vivo* and *in vitro*. Generally, VM structures are positive for PAS but negative for CD31 staining in tumor samples [3]. However, studies showed that CD31^−^/PAS^+^ areas are lacking a lumen in the *in vitro* experiments [21]. At least, the IHC staining results obtained from xenograft samples showed recognized VM structures. To illustrate the role of canstatin in glioma-mediated VM formation, we examined the expression of VM-associated factors such as VEGF and HIF-1α, one of the principal drivers of VM [22] in the lentivirally conduced U87 cells overexpressing canstatin. The results indicated that the production of both VEGF and HIF-1α was decreased upon enhancing canstatin expression. These results suggest that canstatin contributes to the elimination of VM or VM-like structures in glioma tissues in addition to its antiangiogenic ability [20].

Studies have shown that the upregulated expression of survivin in tumors is connected to VEGF-induced PI3K/Akt activation [13]. As sruvivin-mediated VEGF production has also been reported, a positive feedback loop was suggested in which VEGF/Akt-induced survivin expression is followed by VEGF secretion [23]. In this study, we revealed that overexpression of canstatin suppressed the activation of Akt, leading to downregulation of VEGF and survivin in U87 cells. As survivin is an apoptosis inhibitor in tumor cells, canstatin might induce glioma cell apoptosis by inhibiting the VEGF/Akt/survivn pathway. Interestingly, both survivin and VEGF expression can be upregulated by hypoxia-inducible factors, including HIF-1α [24]. Our result showed that canstatin overexpression reduced the production of HIF-1α and its downstream MMP-2, which has the hypoxia response elements (HRE) in the gene regulation region [25]. It was reported that VM formation in glioma samples was related to the increased VEGF and MMP-2 [26]. Although the exact mechanism remains unclear, the results suggested an interaction between VEGF and HIF-1α/MMP-2 pathways regulated by canstatin. Thus, canstatin is considered an attractive therapeutic target for the treatment of glioma. Moreover, treatment with canstatin may have fewer side effects than currently approved chemotherapeutic agents because it is an endogenous protein derived from the noncollagenous C-terminal fragment of the type IV collagen α2 chain [7,27].

So far, no clinical study for the tumor therapy using canstatin gene introduction has been yet reported although several preclinical studies have explored varying strategies of canstatin gene delivery. Viral-based vectors provide an efficient means for the modification of eukaryotic cells both in lab research and clinical applications. For instance, adenoviral-mediated gene transfer of canstatin-human serum albumin fusion protein (CanHSA) was performed to evaluate the validity of treatment against tumors in mouse models, including xenografts of MDA-MB-231 breast cancer cells and PC-3 cells [28]. In addition to adenoviral vectors, lentiviral vectors, which are derived from the human immunodeficiency virus, have been extensively investigated and optimized over the past decades [29]. Recently, self-inactivating lentiviral vectors have been used in multiple clinical trials by introducing genes into hematopoietic stem cells [30] and T cells [31]. As of now, cancer cell vaccines using lentiviral vectors have been investigated [32]. Our study provides a theoretical basis for developing a genetically engineered canstatin therapy using lentiviral vectors to treat gliomas. In the further work, a number of issues when using this approach must be addressed, including efficiency, immunogenicity, and tissue specificity.

## 5 Conclusion

This study demonstrated that canstatin overexpression can inhibit glioma growth and VM-like structure formation, which is correlated with reduced expression of VEGF and HIF-1α. VEGF/Akt/surviving and HIF-1α/MMP-2 pathways might be involved in canstatin-inhibited VM formation in U87 glioma cells.
